# Comparative analysis of optogenetic actuators in cultured astrocytes

**DOI:** 10.1016/j.ceca.2014.07.007

**Published:** 2014-09

**Authors:** Melina Figueiredo, Samantha Lane, Randy F. Stout, Beihui Liu, Vladimir Parpura, Anja G. Teschemacher, Sergey Kasparov

**Affiliations:** a School of Physiology and Pharmacology, School of Medical Sciences, University of Bristol, BS8 1TD, UK; b Department of Neurobiology, Center for Glial Biology in Medicine, University of Alabama, Birmingham, AL 35294, USA; c The Dominick P. Purpura Department of Neuroscience, Albert Einstein College of Medicine, Bronx, NY 10461, USA; d Department of Biotechnology, University of Rijeka, 51000 Rijeka, Croatia

**Keywords:** Astrocyte, ChR2, CatCh, OptoARs, Calcium

## Abstract

Astrocytes modulate synaptic transmission via release of gliotransmitters such as ATP, glutamate, d-serine and l-lactate. One of the main problems when studying the role of astrocytes *in vitro* and *in vivo* is the lack of suitable tools for their selective activation. Optogenetic actuators can be used to manipulate astrocytic activity by expression of variants of channelrhodopsin-2 (ChR2) or other optogenetic actuators with the aim to initiate intracellular events such as intracellular Ca^2+^ ([Ca^2+^]_*i*_) and/or cAMP increases. We have developed an array of adenoviral vectors (AVV) with ChR2-like actuators, including an enhanced ChR2 mutant (H134R), and a mutant with improved Ca^2+^ permeability (Ca^2+^ translocating channelrhodopsin, CatCh). We show here that [Ca^2+^]_*i*_ elevations evoked by ChR2(H134R) and CatCh in astrocytes are largely due to release of Ca^2+^ from the intracellular stores. The autocrine action of ATP which is released under these conditions and acts on the P2Y receptors also contributes to the [Ca^2+^]_*i*_ elevations. We also studied effects evoked using light-sensitive G-protein coupled receptors (opto-adrenoceptors). Activation of optoα1AR (Gq-coupled) and optoβ2AR (G_*s*_-coupled) resulted in astrocytic [Ca^2+^]_*i*_ increases which were suppressed by blocking the corresponding intracellular signalling cascade (phospholipase C and adenylate cyclase, respectively). Interestingly, the bulk of [Ca^2+^]_*i*_ responses evoked using either optoAR was blocked by an ATP degrading enzyme, apyrase, or a P2Y1 receptor blocker, MRS 2179, indicating that they are to a large extent triggered by the autocrine action of ATP. We conclude that, whilst optimal tools for control of astrocytes are yet to be generated, the currently available optogenetic actuators successfully initiate biologically relevant signalling events in astrocytes.

## Highlights

• We compared six optogenetic tools to selectively stimulate and study astrocytes.
• Channelrhodopsin-2 variants cause release of Ca^2+^ from intracellular stores.
• Opto-GPCRs activate selective second messenger cascades, leading to [Ca^2+^]_*i*_ rises.
• Autocrine action of ATP mediates the bulk of [Ca^2+^]_*i*_ signals evoked by opto-GPCRs.
• Current optogenetic tools initiate relevant signalling events in astrocytes.

## 1 Introduction

There is a consensus that astrocytes play a major role in the physiology of the brain and are actively processing information rather than passively witnessing the working of neurones. For decades the major obstacle for studies into the roles of astrocytes was the lack of tools suitable for selective manipulation of these cells, *in situ*, in the context of intact brain tissue. Various genetic tools targeted to astrocytes have allowed a change in the situation. Optogenetic actuators were initially introduced into neuroscience with the aim of controlling neuronal activity, but it also turned out to be possible to manipulate astrocytes by expression of channelrhodopsin-2 (ChR2) or closely related proteins [1], [2]. Light-gated ion channels are introduced into neurones in order to control their membrane potential by light, because these proteins permit cations to cross the plasma membrane leading to depolarization. In the simplest scenario this allows triggering of action potentials by flashes of blue light [3]. However, in the case of astrocytes, such rapid oscillations of membrane potential appear meaningless as they do not emulate any known physiological mechanism of astrocytic activation. Communication to and between astrocytes is largely mediated by an array of G-protein-coupled receptors (GPCRs). Yet, light sensitive ion channels may still be used to initiate intracellular events reminiscent of the metabolic activation of astrocytes (for example, release of Ca^2+^ from intracellular stores or increase in cAMP or glycogenolysis) or to trigger the release of signalling molecules from astrocytes, such as ATP [4], glutamate [2], [5], [6], [7] or l-lactate [8]. How exactly ChR2 leads to Ca^2+^ increases in astrocytes is not clear. Usually, it was assumed that Ca^2+^ ions enter the cytosol of the cell from the extracellular space because ChR2 has low, but detectable, Ca^2+^ permeability [9], [10]. We have generated an array of viral vectors with variants of ChR2 and re-evaluated this issue using both the enhanced ChR2 mutant (H134R) [11] and a more recent mutant with enhanced Ca^2+^ permeability termed Ca^2+^ translocating channelrhodopsin (CatCh) [12]. Specifically, we have tested whether Ca^2+^ elevations evoked by ChR2(H134R) in astrocytes involve release of Ca^2+^ from intracellular stores and whether autocrine action of ATP, which is secreted under these conditions, plays a role. Indeed, it is plausible that ATP released by light stimulation [13] acts on purinergic receptors that are highly expressed in astrocytes.

Since GPCR activation is more in line with the normal physiological functioning of astrocytes, light-sensitive GPCRs (opto-adrenoceptors; optoARs), generated by fusing components of rhodopsin and either α_1_- or β_2_-adrenergic receptors are, perhaps, a more suitable alternative for astrocyte optogenetics [14]. These chimaeras were designed to couple to the same intracellular pathways as the native α_1_- and β_2_-adrenergic receptors, which in theory should permit selective activation by light of either G_*q*_- or G_*s*_-coupled cascades in astrocytes. In this study, we investigated the effects evoked in cultured astrocytes by these tools and compared them to those of ChR2 variants.

## 2 Materials and methods

### 2.1 Primary cultures of rat astrocytes

Primary cultures of astrocytes were prepared from the cerebral cortices, cerebellum and brainstem of Wistar rat pups (P2) following protocols adapted from [15] as described previously [13].

Briefly, the brains of Wistar P2 pups were dissected out, crudely cross-chopped and bathed in a solution containing Hank's Balanced Salt Solution (HBSS; Invitrogen), deoxyribonuclease I from bovine pancreas (0.04 mg/ml of DNase I; Sigma), trypsin from bovine pancreas (0.25 mg/ml; Sigma) and bovine serum albumin (3 mg/ml of BSA; Sigma). The preparation was agitated at 37 °C for 15 min. Trypsination of the brain tissue was terminated by the addition of equal volumes of culture media comprised of Dulbecco's Modified Eagle Medium (DMEM; Invitrogen), 10% heat-inactivated Foetal Bovine Serum (FBS; Invitrogen), 100 U/ml penicillin and 0.1 mg/ml streptomycin (P/S; Invitrogen), and then centrifuged at 2000 rpm, at room temperature for 10 min. The supernatant was aspirated off and the remaining pellet was resuspended twice with a 15 ml and 5 ml solution containing HBSS, BSA (3 mg/ml) and DNase I (0.04 mg/ml). After the cell debris had settled, the cell suspension was filtered (40 μm cell strainer, BD Falcon) and centrifuged (2000 rpm, 5 min, 20 °C). Cells were seeded in a T75 flask (Corning) containing culture media and maintained at 37 °C with 5% CO_2._ Once the cultures reached confluence, the flasks were mildly shaken overnight and media were exchanged to remove microglia and oligodendrocytes.

### 2.2 Construction of optogenetic actuator vectors

In this study, we compared the impact on astrocytic [Ca^2+^]_*i*_ handling of four light-sensitive ion channel constructs and two light-activated GPCRs: ChR2-Venus, a channelrhodopsin-2 fusion with the yellow fluorescent protein Venus; ChR2(H134R)-Katushka1.3, an enhanced channelrhodopsin-2 mutant fused to the far red-shifted fluorescent protein Katushka1.3 [4], [11]; ChR2(H134R)-mKate, a fusion with a monomeric version of Katushka [16]; CatCh-EYFP, a hChR2(L132C) mutant of channelrhodopsin-2 with increased Ca^2+^ permeability fused to enhanced yellow fluorescent protein [12]; optoα_1_AR-YFP and optoβ_2_AR-YFP, chimaeras of opsin-α_1_- and opsin-β_2_-adrenergic receptors, respectively, fused to yellow fluorescent protein [8], [14]. CatCh was constructed by mutagenesis from hChR2(H134R) using the InFusion 2 PCR-cloning kit (Clontech Laboratories, Mountainview, CA) with the following primers:

VectorFwd-TCACTTGTCCTGTCATCTGTATCCACCTGAGC
VectorBwd-TAACATTGATCCTCAGGGACCAGGACGG
InsertFwd-TGAGGATCAATGTTACTGTGCCGGATGG
InsertBwd-TGACAGGACAAGTGAGCAGCCACTCTGC

Primers generated the L132C mutation (and reverted to R134H) when used with the template pcDNA3.1v5his-hChR2(H134R)-EYFP which was kindly provided by Prof. Karl Deisseroth (Stanford University, Stanford, CA). Optoα_1_AR-YFP and optoβ_2_AR-YFP clones were also kindly provided by K. Deisseroth. All constructs were subcloned into adenoviral shuttle plasmids (pXCX) containing a shortened promoter of the human glial fibrillary acidic protein (GfaABC1D; 694-bp) [17]. A two-step transcriptional amplification strategy (TAS) was used to enhance transgene expression driven by GfaABC1D [18], [19]. This promoter is abbreviated as sGFAP within the transgene constructs. Adenoviral vectors (AVV) were produced by spontaneous recombination of the shuttle plasmids and the helper plasmid (pBHG10), as previously described [20].

Optogenetic constructs are typically expressed in target cells using strong promoters and multiple copies of viral genomes to achieve high level of expression. We estimated the potential toxicity of overexpression of these biologically active proteins by comparing cultured astrocytes, transduced with optogenetic and control (enhanced green fluorescent protein - EGFP) AVV, at a range of titres (10^6^–10^10^ TU/ml) for signs of deterioration (i.e., swollen cells, multinuclear cellular agglomerates and large areas with no cells present). Transduced astrocytes were daily inspected for signs of deterioration which, if present, were usually evident already after 24 h. All ChR2-derived constructs and EGFP-only expressing AVV caused no visible change in the density or overall appearance of astrocytes when used in titres 10^6^ TU/ml or 10^7^ TU/ml. Viral vectors driving the expression of optoα_1_AR and optoβ_2_AR did not evoke any visible detrimental effects when used in titres up to 10^8^ TU/ml. Higher titres of all AVV, including the EGFP control, had similar negative effects on morphology and density of astrocytes (Supplementary Fig. S1).

Supplementary material related to this article can be found, in the online version, at http://dx.doi.org/10.1016/j.ceca.2014.07.007.

Supplementary Fig. I Detrimental effects on astrocytes in culture caused by AVV. Signs of deterioration caused by toxic titres of different AVV were evident in bright field as soon as 24 h after the transduction of astrocytes. (A) AVV-sGFAP-ChR2-Venus (24 h; 4 × 10^8^ TU/ml). (B) AVV-sGFAP-ChR2(H134R)-Katushka1.3 (24 h; 1 × 10^9^ TU/ml). (C) AVV-sGFAP-ChR2(H134R)-mKate (24 h; 4 × 10^8^ TU/ml). (D) AVV-sGFAP-CatCh-EYFP (24 h; 5.6 × 10^8^ TU/ml). (E) AVV-sGFAP-optoβ2AR (24 h; 1.9 × 10^10^ TU/ml). (F) AVV-sGFAP-optoα1AR (24 h; 2.6 × 10^10^ TU/ml). (G) AVV-sGFAP-EGFP (24 h; 4.8 × 10^8^ TU/ml). (H) AVV-sGFAP-EGFP (healthy astrocytes; 24 h; 4.8 × 10^7^ TU/ml).

### 2.3 Optogenetic stimulation and [Ca^2+^]_*i*_ imaging in cultured astrocytes

All optogenetic constructs used here are optimally activated by blue light and have low sensitivity to yellow light. In order to avoid optogenetic stimulation during [Ca^2+^]_*i*_ imaging, we used the Ca^2+^ indicator Rhod-2 AM (Biotium) which can be excited by the green or yellow laser of the confocal microscope. Primary cultures of rat astrocytes seeded onto 13 mm coverslips and transduced with AVV were loaded with Rhod-2 AM 1 h prior to experimentation. Confocal imaging was carried out at 34 °C under continuous superfusion with HBSS (pH 7.4) in a chamber mounted on an SP2 Leica confocal microscope. Images were acquired through a water immersion objective (40×) using the 561 nm laser set to low power (<20% of the line power) in a time-lapse mode to excite Rhod-2. We confirmed that this excitation regime was unable to activate the optogenetic constructs during the very brief scans. Activation of optogenetic constructs was achieved with moderate intensity blue light (470 nm laser diode microscope illumination system from Rapp OptoElectronic, Germany). In order to illuminate the cells with blue light and at the same time continue imaging Rhod-2, we modified the light path of the confocal microscope and added an additional dichroic mirror [13]. We aimed to compare effects of light stimulation and drugs on cultured astrocytes within the same coverslip. At the same time we wanted to avoid repetitive activations of the same cohort of cells in order to minimise a possible impact of desensitisation or depletion of intracellular Ca^2+^ stores. Hence, after each stimulation episode the objective was moved to a new field of view in a different part of the coverslip.

### 2.4 Drugs

Drugs were purchased from either Sigma (apyrase, thapsigargin, cyclopiazonic acid – CPA) or Tocris Bioscience (MRS 2179, SQ22536 and U73122). Two approaches were used to investigate involvement of purinergic autoreceptors on astrocytes. MRS 2179 is widely considered to be a selective antagonist of metabotropic P2Y1 receptors, but may also affect P2X1 and P2X3 receptors [21]. Apyrase is an ATP-degrading enzyme which can be used to non-discriminately block the actions of extracellular ATP. Apyrase was applied for 30 min before light-stimulation of astrocytes.

### 2.5 Data analysis

[Ca^2+^]_*i*_ responses in individual astrocytes were assessed using Leica software and expressed as changes in relative fluorescence intensity (*F*/*F*_0_, where *F*_0_ is the baseline fluorescence of a cell). Further statistical analysis was carried out using Excel and/or Graphpad Prism^®^ software. A typical fleld of view contained 10–20 cells. Cells with unstable baseline fluorescence (e.g. significant stochastic changes in fluorescence before application of drugs or light stimulation) were excluded from the analysis.

## 3 Results

### 3.1 Comparison of ChR2 and its mutants as tools to trigger [Ca^2+^]_*i*_ increases in cultured astrocytes

**Fig. 1:**
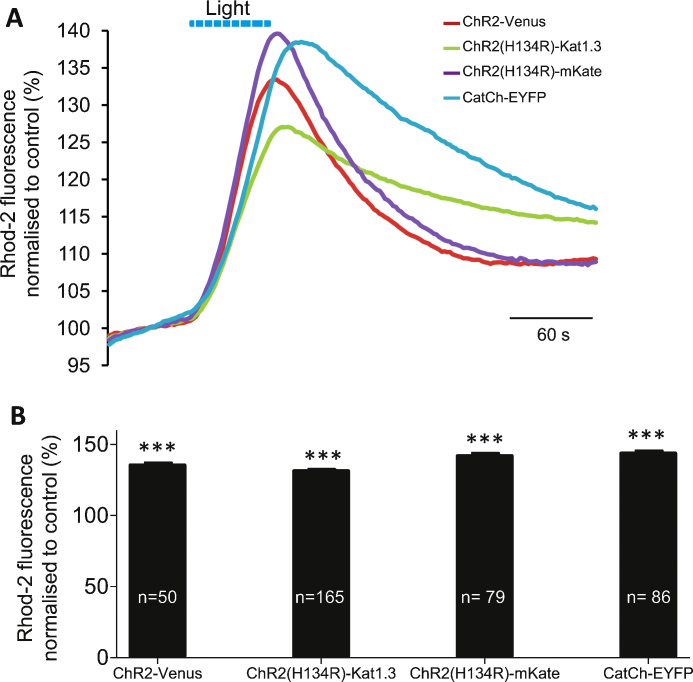
Comparison of ChR2 and its mutants as tools to evoke [Ca^2+^]*_i_* elevations in cultured astrocytes. (A) Averaged normalised traces recorded in response to light stimulation in astrocytes transduced with different optogenetic actuators: AVV-sGFAP-ChR2-Venus (red; *n* = 50); AVV-sGFAP-ChR2(H134R)-Katushka1.3 (green, *n* = 165); AVV-sGFAP-ChR2(H134R)-mKate (purple, *n* = 79); AVV-sGFAP-CatCh-EYFP (blue, *n* = 86). (B) Summary statistics for peak values of light-induced [Ca^2+^]*_i_* elevations. Student's paired *t*-test was carried out against own baseline. Each column represents cellular responses pooled from at least 3 independent experiments. ***p < 0.01. (For interpretation of the references to colour in this figure legend, the reader is referred to the web version of the article.)

Astrocytes expressing ChR2-Venus, ChR2(H134R)-Katushka1.3, ChR2(H134R)-mKate and CatCh-EYFP were loaded with Rhod-2 AM and stimulated with flashes of blue light (20/20 ms duty cycle) for 60 s. Stimulation with the same intensity of light evoked highly significant [Ca^2+^]_*i*_ increases which were of comparable magnitude between astrocytes expressing the different ChR2 variants (Fig. 1A and B). Since the absolute amplitude of the [Ca^2+^]_*i*_ response depends on both, the properties of the construct and its level of expression in the cell (which is limited by potential toxicity and also the ability to deliver the construct to the relevant membranes), this parameter should not be directly interpreted as a measure of the “power” of individual actuators. At the same time, the latency of [Ca^2+^]_*i*_ responses and their overall dynamics were similar between all tested ChR2 variants.

### 3.2 Autocrine action of ATP contributes to the [Ca^2+^]_*i*_ increases in ChR2(H134R)- and CatCh-expressing astrocytes

**Fig. 2:**
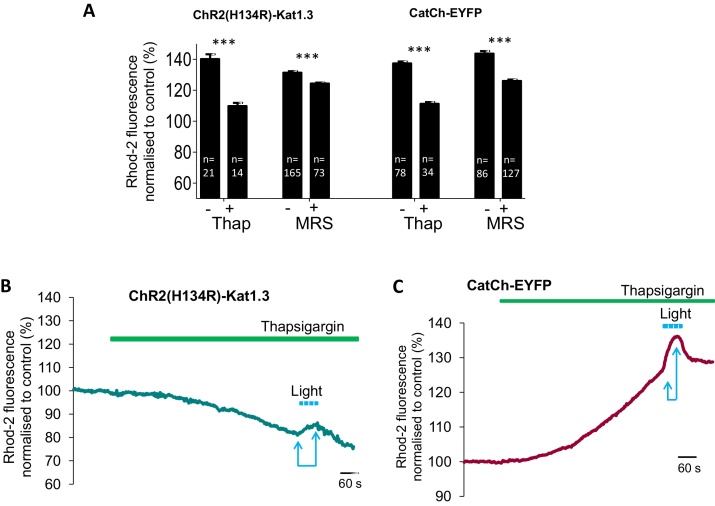
[Ca^2+^]*_i_* elevations triggered by ChR2-derived actuators depend on intracellular Ca^2+^ mobilisation and autocrine ATP action. (A) Pooled summary data for peak [Ca^2+^]*_i_* responses to light stimulation in ChR2(H134R)-Katushka1.3 or CatCh-EYFP-expressing astrocytes. In presence of MRS 2179 (MRS; 10 μM) and thapsigargin (Thap; 1 μM), [Ca^2+^]*_i_* elevations were significantly reduced as compared to control.***p < 0.0001, Student's unpaired *t*-test. (B, C) Pre-treatment with thapsigargin (1 μM) for 10 min decreased the light-induced [Ca^2+^]*_i_* increases in astrocytes expressing ChR2(H134R)-Katushka1.3 and CatCh-EYFP. Averaged traces normalised to their own baselines. Arrows indicate epochs used for calculation of values in A. Note slow rise in intracellular Ca^2+^ in CatCh-expressing cells during application of thapsigargin, this effect was highly reproducible.

ChR2(H134R) can trigger release of ATP from astrocytes [13] which is a potent stimulant of Ca^2+^ release from stores in these cells. Application of MRS 2179 (10 μM), a P2Y1 receptor antagonist, significantly reduced the light-induced [Ca^2+^]_*i*_ increases in both ChR2(H134R) and CatCh-expressing astrocytes (Fig. 2A), indicating an autocrine action of ATP.

### 3.3 [Ca^2+^]_*i*_ increases in ChR2(H134R)- and CatCh-expressing astrocytes are mainly due to release from intracellular stores

It is generally assumed that [Ca^2+^]_*i*_ elevations registered in this and previous studies are due to the influx of extracellular Ca^2+^ ions via the plasma membrane of the astrocytes. We have tested the alternative hypothesis that activation of ChR2(H134R) and CatCh triggers the release of Ca^2+^ from intracellular stores. Thapsigargin (1 μM), a potent inhibitor of the ATP-dependent Ca^2+^ pump of endoplasmic reticulum (ER), despite the presence of normal concentrations of Ca^2+^ in the extracellular media, almost completely blocked [Ca^2+^]_*i*_ elevations in astrocytes expressing ChR2(H134R) (Fig. 2B) and CatCh (Fig. 2C) in response to light stimulation (summary statistics are shown in Fig. 2A). Thus, with both ChR2(H134R) and CatCh, the bulk of the Ca^2+^ rise in the cytoplasm of the stimulated astrocytes is derived from thapsigargin-sensitive intracellular compartments. Interestingly, application of thapsigargin to astrocytes expressing CatCh led to a slow build-up of [Ca^2+^]_*i*_ even in the absence of blue light stimulation (Fig. 2C). This was not observed in cells expressing ChR2(H134R) (Fig. 2B). CPA (10 μM), an inhibitor of the sacroplasmatic reticulum Ca^2+^ pump, also potently suppressed Ca^2+^ elevations evoked by light stimulation of ChR2(H134R)-expressing astrocytes (*p* < 0.001, *n* = 60, data not shown).

### 3.4 Optoα1AR signals via phospholipase C in astrocytes

**Fig. 3:**
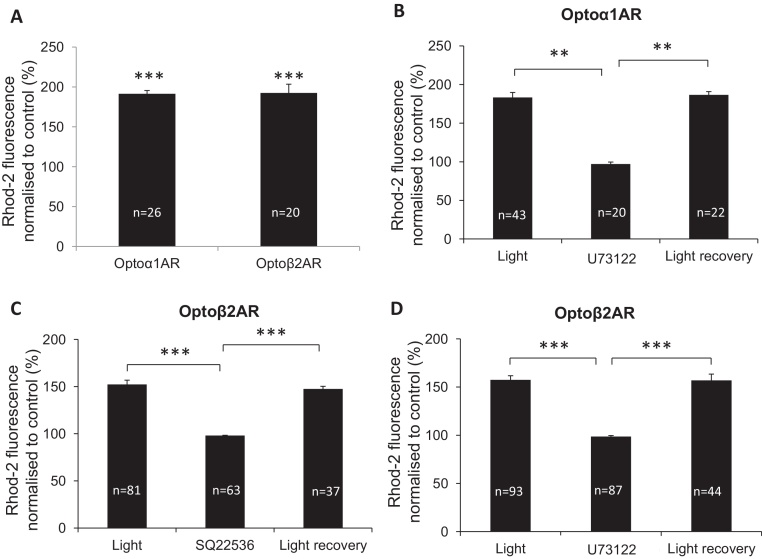
[Ca^2+^]*_i_* elevations in astrocytes in response to optoAR stimulation are mediated by specific intracellular second messenger cascades. (A) Average increases in [Ca^2+^]*_i_* in responding stimulated astrocytes expressing optoα1AR and optoβ2AR. ***p < 0.0001 compared to own baselines, Student's paired *t*-test. Pooled data from at least 3 independent experiments in each case. (B) The PLC antagonist U73122 (10 μM) completely and reversible abolished light-induced [Ca^2+^]*_i_* increases in astrocytes expressing optoα_1_AR. **p < 0.001, Student's unpaired *t*-test. (C) The adenylate cyclase inhibitor SQ22536 (100 μM) completely abolished the light-induced [Ca^2+^]*_i_* increases in astrocytes expressing optoβ_2_AR. ***p < 0.0001, Student's unpaired *t*-test. (D) [Ca^2+^]*_i_* elevations in optoβ2AR-expressing astrocytes were completely blocked by U73122 (10 μM). ***p < 0.0001, Student's unpaired *t*-test.

Light stimulation of optoα_1_AR-expressing and Rhod-2 AM-loaded astrocytes generated [Ca^2+^]_*i*_ elevations (Fig. 3A). To verify that optoα_1_AR recruits the expected Gq-protein-mediated intrinsic signalling pathway in astrocytes, we used an antagonist of phospholipase C (PLC), U73122 (10 μM), which completely and reversibly abolished light-induced fluorescence increases (Fig. 3B).

### 3.5 Optoβ2AR signals via adenylate cyclase in astrocytes

In astrocytes transduced with optoβ2AR which are expected to couple to Gs proteins, light stimulation, surprisingly, also generated [Ca^2+^]_*i*_ elevations (Fig. 3A). To verify that optoβ_2_AR recruits the G_*s*_-signalling cascade we used an adenylate cyclase blocker SQ22536 (100 μM) which completely suppressed all responses (Fig. 3C). The effect of SQ22536 was reversible. [Ca^2+^]_*i*_ elevations induced using optoβ2AR stimulation were also completely blocked by the PLC antagonist U73122 (10 μM) (Fig. 3D).

### 3.6 Autocrine action of ATP mediates the bulk of [Ca^2+^]_*i*_ increases when astrocytes are activated using optoARs

**Fig. 4:**
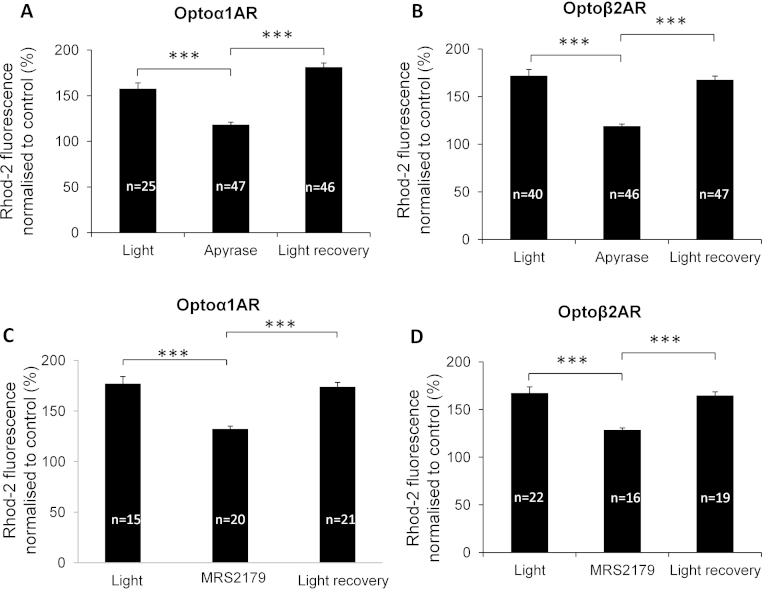
Autocrine action of ATP is responsible for the bulk of the [Ca^2+^]*_i_* elevations evoked using optoARs. (A, B) Extracellular pre-treatment with the ATP degrading enzyme apyrase strongly attenuated [Ca^2+^]*_i_* responses triggered by light in optoα_1_AR- and optoβ_2_AR-expressing astrocytes. (C, D) MRS 2179 significantly attenuated [Ca^2+^]*_i_* responses evoked by light stimulation of optoα_1_AR- and optoβ2AR-expressing astrocytes. ***p < 0.0001, Student's unpaired *t*-test.

In order to investigate whether responses evoked in optoAR-expressing astrocytes are driven by the autocrine action of ATP, we pre-treated cultures with the ATP-degrading enzyme apyrase. Apyrase drastically reduced light-induced [Ca^2+^]_*i*_ increases in astrocytes expressing either optoα_1_AR or optoβ_2_AR (Fig. 4A and B). Similar results were obtained using MRS 2179 (10 μM), which significantly attenuated [Ca^2+^]_*i*_ increases in astrocytes expressing either optoα_1_AR or optoβ_2_AR by approximately 40% (Fig. 4C and D). Effects of MRS 2179 and apyrase were reversible.

## 4 Discussion

There is a consensus that astrocytes play a major role in the physiology of the brain and contribute to information processing via modulation of neuronal functions. In order to better understand the roles which these cells play, we need tools which allow us to selectively control their activity and, ideally, mimic their physiological activation. Traditionally, communication between different cell types in the brain was studied using electrophysiological and pharmacological approaches. These techniques, although useful, have many limitations. Electrical stimulation of astrocytes is hardly meaningful, while drugs designed to understand the role of an intracellular pathway are hardly ever cell-specific.

In order to overcome this problem, previous studies used sophisticated molecular approaches, for example ectopic expression in astrocytes of the receptor Mrg1 in astrocytes [22], [23] or an artificial designer GPCR [24].

Our laboratory has previously employed the ChR2 variant ChR2(H134R) to activate astrocytes located within the central chemosensitive area, the Retrotrapezoid Nucleus [13]. It was noticed that, upon activation, ChR2(H134R) triggered [Ca^2+^]_*i*_ increases in astrocytes. Similar observations were also made by several other groups [1], [7]. However, how exactly activation of ChR2 or similar actuators leads to this [Ca^2+^]_*i*_ increase remained unclear. It has been suggested that light-induced [Ca^2+^]_*i*_ increases may be evoked by Ca^2+^ influx from the extracellular space [4] or Na^+^ entry through ChR2, followed by the reverse activity of the Na^+^/Ca^2+^ exchanger.

Therefore, one of the aims of this study was to re-evaluate this issue using both the enhanced ChR2 mutant (H134R) [11] and, for comparison, a more recently developed mutant with enhanced Ca^2+^ permeability, CatCh [12]. It was noticeable that CatCh was not more potent, in terms of speed or amplitude of responses, or conferred any other advantage. Using thapsigargin, we demonstrate here that Ca^2+^ elevations evoked by both ChR2(H134R) and CatCh in astrocytes are largely due to the release of Ca^2+^ from the intracellular stores. A recent study from the M. Sur and E. Boyden laboratories [1] came to a similar conclusion. Curiously, in CatCh-transduced astrocytes, even without light stimulation thapsigargin caused a progressive slow build up in [Ca^2+^]_*i*_ over the course of several minutes. This phenomenon was highly reproducible and suggests that CatCh may affect Ca^2+^ handling even without being activated. At present we do not know the mechanism of this effect, but it might indicate an additional stress on ER by this protein.

The significant reduction in [Ca^2+^]_*i*_ responses in the presence of MRS 2179 is consistent with the idea that stimulated astrocytes release ATP, which then acts in an autocrine/paracrine manner, at least under *in vitro* conditions (Fig. 2A). It follows that in tissue with high activity of ectonucleotidases, responses to ChR2 could be greatly reduced due to the faster inactivation of ATP. It is worth to notice that expression of ATP-degrading enzymes indeed may be radically different between mice and rats [25] at least in some parts of the central nervous system.

Although stimulation of ChR2 with blue light triggers ATP release, stimulates l-lactate production and release [8], and evokes [Ca^2+^]_*i*_ responses in astrocytes, it does not mimic any endogenous mechanism of astrocyte activation, this being an obvious issue with the interpretation of the results. Communication to and between astrocytes is generally thought to be mediated mainly by an array of GPCRs, although other mechanisms such as coupling via local ion gradients and by gap junctions have recently been discovered [26]. Thus, the second aim of this study was to evaluate optogenetic tools which control astrocytes by selective recruitment of intrinsic signalling cascades. In contrast to ChR2 and CatCh, the GPCR chimaeras tangibly mimic the events evoked by a neurotransmitter such as noradrenaline binding to the native α_1_AR and β_2_AR. Therefore, such tools in theory provide a better way to optically control astrocytes. While it could be expected that activation of a Gq-coupled receptor should result in an increase in [Ca^2+^]_*i*_, we were surprised to see the same when using the optoβ2AR construct (Fig. 3A). Nevertheless, in both cases blocking the early steps of the intracellular signalling cascades (PLC in case of optoα1AR and adenylate cyclase in case of optoβ2AR) abolished Ca^2+^ elevations evoked using these artificial receptors, confirming that they do couple to the predicted targets. However, our results suggests that the [Ca^2+^]_*i*_ increases evoked by using both actuators are, in fact, largely due to the autocrine action of ATP (Fig. 4). The residual response remaining after apyrase and MRS 2179 treatment (average increases in *F*/*F*_0_ by ∼20%) was too small to further reliably study, but could represent the “pure” effect of optoα1AR signalling. In the case of optoβ2AR, it is possible that the initial cAMP mediated step then leads to cross-activation of PLC via a previously established link mediated by exchange protein activated by cAMP (EPAC) [27], [28], [29], [30].

Of note, repeated stimulation produced highly variable responses in the same astrocytes and for this reason, the effects of drugs were evaluated on cells only exposed to light once and therefore imaged in distinct areas of the same coverslip. A recent study found that cAMP responses induced by optoβ2AR are strongly affected by photobleaching which these constructs have inherited from their progenitor protein, rhodopsin. By replacing mammalian opsin sequences with those derived from an invertebrate jellyfish *Carybdea rastonii*, a substantial increase in the performance of the construct (JellyOp) was achieved [31]. We would anticipate that this new tool will offer significant advantages for control of cAMP mediated signalling in astrocytes. Interestingly, we hardly ever observed any visible fluorescence in cells transduced with optoARs even though both are tagged with YFP. We speculate that there may be a mechanism which caps the expression level of GPCRs in the transduced cells, possibly via targeted degradation. This feature complicates the use of these constructs in imaging experiments because the expressing cells cannot be identified in real time.

Finally, our comparison of the survival rates of cultured astrocytes indicated that all AVV driving the expression of ChR2, ChR2(H134R), CatCh and EGFP (the latter known as fairly biologically inert) caused major cell damage and toxicity at titres in excess of 10^8^ TU/ml (Supplementary Fig. S1), suggesting that the damage is related to an excessive AVV load rather than a specific feature of any of the heterologously expressed proteins.

## 5 Conclusions

Comparison of various optogenetic actuators in this study has revealed that all of them share a common signalling effect in astrocytes, culminating in release of their main signalling molecule, ATP. Autocrine action of ATP plays a major role in the [Ca^2+^]_*i*_ elevations induced with both light-sensitive ion channels and optoARs. In terms of the ability to induce [Ca^2+^]_*i*_ responses we did not detect any radical differences between ChR2, ChR2(H134R), CatCh or optoARs. Surprisingly, [Ca^2+^]_*i*_ elevations induced by ChR2(H134R) and CatCh depend almost entirely on Ca^2+^ release from thapsigargin-sensitive intracellular stores, possibly due to the retention of these proteins in the endomembranes. Thus, in terms of end point effects (release of ATP and l-lactate [8]), ChR2 derivatives are similar to optoARs. While they are more user friendly, the downside of the use of these ion channels are the inevitable membrane depolarisations they evoke which might be seen as non-physiological in astrocytes. We conclude that the currently available optogenetic constructs can be employed to initiate robust signalling events in astrocytes but, clearly, optimal tools for optical control of astrocytic activity are yet to be generated.

## Author contributions

M.F. performed the optogenetic experiments with astrocytes expressing ChR2 (and its mutants) and the experiments to assess the toxicity of the viral vectors, generated some of the AVVs, performed data analysis of the optogenetic experiments with ChR2, prepared some of the figures and wrote some parts of the manuscript. S.L. performed the experiments with optoARs, generated some of the AVVs, prepared some of the figures, performed some of the data analysis. V.P. and R.F.S. Jr. synthesised and provided CatCh. B.H.L. generated some of the AVV and also helped M.F. and S.L. with the design of some of the constructs. A.G.T. guided both M.F. and S.L. with AVV and study design, and edited the text and figures. S.K. designed the study, wrote parts of the manuscript, edited figures and text.
