# Supplementary material for: Comparative analysis of optogenetic actuators in cultured astrocytes
Source: Cell Calcium. 2014 Sep;56(3):208–14. doi: 10.1016/j.ceca.2014.07.007 (PMC4169180; doi:10.1016/j.ceca.2014.07.007)
Supplement: Supplementary Fig. I — Detrimental effects on astrocytes in culture caused by AVV. Signs of deterioration caused by toxic titres of different AVV were evident in bright field as soon as 24 h after the transduction of astrocytes. (A) AVV-sGFAP-ChR2-Venus (24 h; 4 × 10^8^ TU/ml). (B) AVV-sGFAP-ChR2(H134R)-Katushka1.3 (24 h; 1 × 10^9^ TU/ml). (C) AVV-sGFAP-ChR2(H134R)-mKate (24 h; 4 × 10^8^ TU/ml). (D) AVV-sGFAP-CatCh-EYFP (24 h; 5.6 × 10^8^ TU/ml). (E) AVV-sGFAP-optoβ2AR (24 h; 1.9 × 10^10^ TU/ml). (F) AVV-sGFAP-optoα1AR (24 h; 2.6 × 10^10^ TU/ml). (G) AVV-sGFAP-EGFP (24 h; 4.8 × 10^8^ TU/ml). (H) AVV-sGFAP-EGFP (healthy astrocytes; 24 h; 4.8 × 10^7^ TU/ml). [file mmc1.pdf]

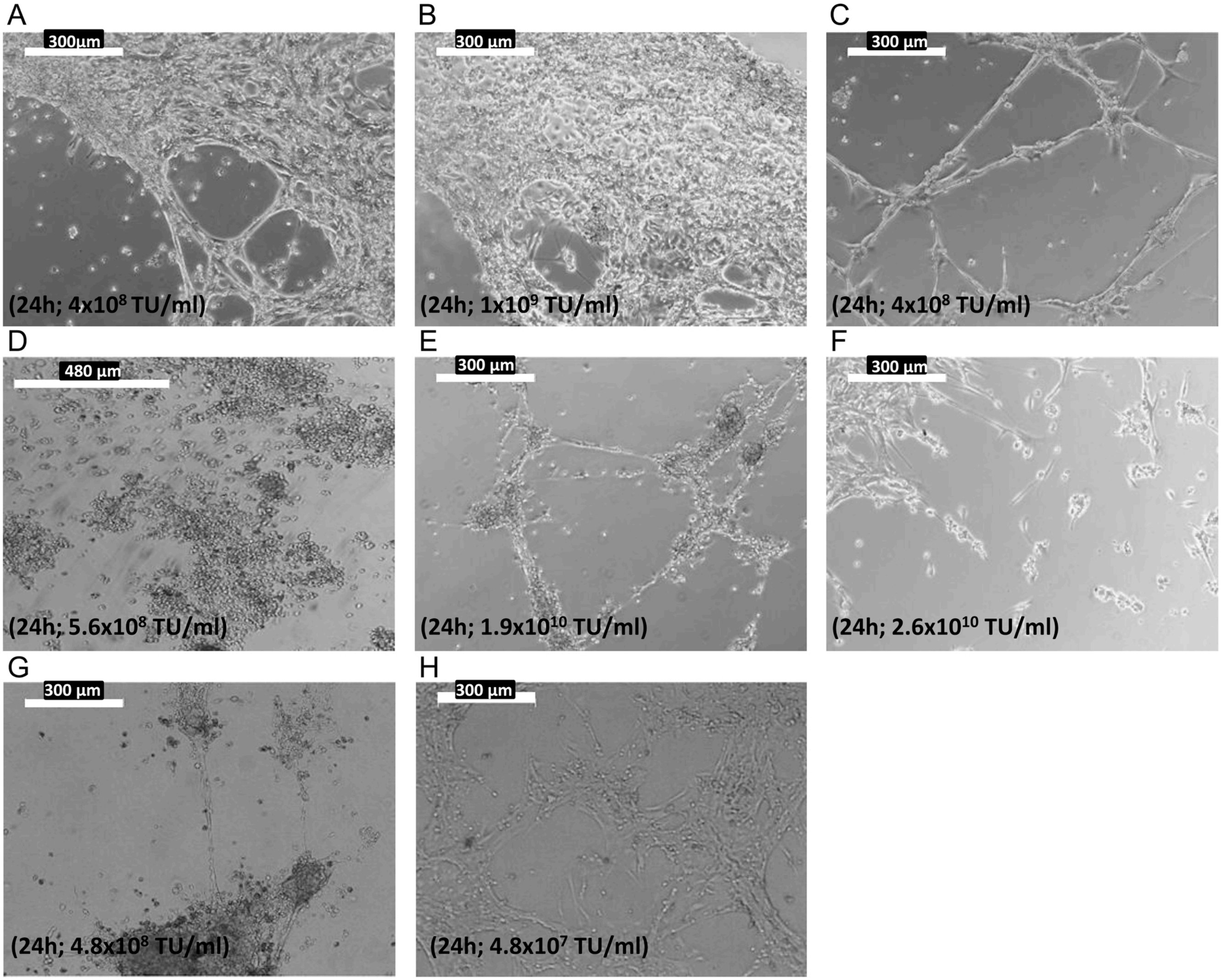

Figure S1

Detrimental effects on astrocytes in culture caused by AVV.

Signs of deterioration caused by toxic titres of different AVV were evident in bright field as soon as 24 hours after the transduction of astrocytes.

A: AVV-sGFAP-ChR2-Venus (24h; 4×10^{8} TU/ml). **B:** AVV-sGFAP-ChR2(H134R)-Katushka1.3 (24h; 1×10^{9} TU/ml). **C:** AVV-sGFAP-ChR2(H134R)-mKate (24h; 4×10^{8} TU/ml). **D:** AVV-sGFAP-CatCh-EYFP (24h; 5.6×10^{8} TU/ml). **E:** AVV-sGFAP-optoβ2AR (24h; 1.9×10^{10} TU/ml). **F:** AVV-sGFAP-optoα1AR (24h; 2.6×10^{10} TU/ml). **G:** AVV-sGFAP-EGFP (24h; 4.8×10^{8} TU/ml). **H:** AVV-sGFAP-EGFP (healthy astrocytes; 24h; 4.8×10^{7} TU/ml).
